# Three IMRT advanced planning tools: A multi‐institutional side‐by‐side comparison

**DOI:** 10.1002/acm2.12679

**Published:** 2019-07-31

**Authors:** Lan Lu, Yang Sheng, Jeremy Donaghue, Zhilei Liu Shen, Matt Kolar, Q. Jackie Wu, Ping Xia

**Affiliations:** ^1^ Department of Radiation Oncology Taussig Cancer Center, Cleveland Clinic Cleveland OH USA; ^2^ Department of Radiation Oncology Duke University Durham NC USA; ^3^ Department of Radiation Oncology Akron General Hospital Akron OH USA

**Keywords:** auto‐planning, knowledge‐based planning, multiple criteria optimization

## Abstract

**Purpose:**

To assess three advanced radiation therapy treatment planning tools on the intensity‐modulated radiation therapy (IMRT) quality and consistency when compared to the clinically approved plans, referred as manual plans, which were planned without using any of these advanced planning tools.

**Materials and Methods:**

Three advanced radiation therapy treatment planning tools, including auto‐planning, knowledge‐based planning, and multiple criteria optimization, were assessed on 20 previously treated clinical cases. Three institutions participated in this study, each with expertise in one of these tools. The twenty cases were retrospectively selected from Cleveland Clinic, including five head‐and‐neck (HN) cases, five brain cases, five prostate with pelvic lymph nodes cases, and five spine cases. A set of general planning objectives and organs‐at‐risk (OAR) dose constraints for each disease site from Cleveland Clinic was shared with other two institutions. A total of 60 IMRT research plans (20 from each institution) were designed with the same beam configuration as in the respective manual plans. For each disease site, detailed isodoseline distributions and dose volume histograms for a randomly selected representative case were compared among the three research plans and manual plan. In addition, dosimetric endpoints of five cases for each site were compared.

**Results:**

Compared to the manual plans, the research plans using advanced tools showed substantial improvement for the HN patient cases, including the maximum dose to the spinal cord and brainstem and mean dose to the parotid glands. For the brain, prostate, and spine cases, the four types of plans were comparable based on dosimetric endpoint comparisons.

**Conclusion:**

With minimal planner interventions, advanced treatment planning tools are clinically useful, producing a plan quality similarly to or better than manual plans, improving plan consistency. For difficult cases such as HN cancer, advanced planning tools can further reduce radiation doses to numerous OARs while delivering adequate dose to the tumor targets.

## 1 INTRODUCTION

The key to intensity‐modulated radiation therapy (IMRT) IMRT planning is the use of a compute optimization to find an optimal balance between delivering adequate prescription dose coverage to the planning target volume (PTV), and sparing normal tissues. Even with compute optimization, finding a clinically optimal IMRT plan for a specific patient is still challenging and time‐consuming, often resulting in large variations in plan qualities among different institutions and planners.^1^, ^2^, ^3^ The large variations in plan quality are partly due to that the manual plan objectives are based on the simplified dose and volume, and not explicitly specified on spatial relationship between the organs‐at‐risk (OARs) and PTVs of the specific patient. Another reason is that most planning optimization algorithms find a solution with a local minimum rather than the global minimum.^4^, ^5^, ^6^ Because of these limitations, progressive adjustments in the planning objectives are often used by experienced planners to create clinical IMRT plans. However, extensive manual progressive adjustments increase the planning time and the outcomes are highly dependent on the planner's skills and experience.

Many research efforts have been devoted to reduce variations in plan quality and accelerate the IMRT planning process, henceforth, several advanced treatment planning tools have been developed and implemented clinically. One method, referred to as the automatic planning (AP), is developed by the Pinnacle treatment planning system (Philips Radiation Oncology Systems, Fitchburg, WI).^7^, ^8^, ^9^ The second method, referred to as the knowledge‐based planning (KBP), is available from the Eclipse treatment planning system (Varian, Palo Alto, CA).^10^, ^11^, ^12^, ^13^ The third approach, named multi‐criteria optimization (MCO) is available in the RaySearch System (Stockholm, Sweden).^14^, ^15^, ^16^

The purpose of this study is to assess advantages of these three advanced treatment planning tools when compared with the clinically accepted IMRT plans without using any of these advanced planning tools. For the remaining text, we refer to these clinical plans as the manual plans. Using the same planning datasets from brain tumor, head‐and‐neck (HN) cancer, advanced prostate cancer with pelvic lymph nodal involvement, and spinal tumors treated with SBRT, three participated institutions applied one of these three advanced planning tools to create IMRT plans with minimum human interventions. We evaluated plan quality of the advanced plans against the manual plans.

## 2 MATERIALS AND METHODS

### 2.1 Study dataset

Twenty clinical cases, including five brain cases, five HN cases, five advanced prostate cases with pelvic lymph nodal involvement, and five spinal tumor metastasis cases treated with SBRT, were retrospectively selected from Cleveland Clinic for this study. The patient identifications were anonymized using MIM software (MIM 6.4, Cleveland, OH) and subsequently sent to the other two participating institutions, Duke University and Akron General Hospital. Three advanced treatment planning tools, including AP, KBP, and MCO, were used in the study. For the KBP approach, the model was trained for each treatment site individually using the in‐house program proposed by Yuan et al.^12^ Each participating institution has clinically implemented one of these advanced planning tools.

For each cancer site, the general planning goals from Clevelan Clinic were sent to the other two institutions without case specific instruction. The manual plans were generated using the Pinnacle planning system according to the same clinical goals at Cleveland Clinic by experienced planners and were clinically approved. IMRT quality assurance measurements for manual plans were conducted and passed the local institution criteria. Table 1 lists the planning guidance for these four sites. Volumetric modulated arc (VMAT) plans were generated for head‐and‐neck, brain and prostate cases and step‐and‐shoot plans were generated for spine cases using these three advanced planning tools, resulting in a total of 60 plans. For each case, all plans used the same beam configurations (Table 2) as in the manual plans, such as the isocenter location, gantry angles for step and shoot plans, number of arcs and arc lengths for VMAT plans, and the collimator angles. All plans were normalized to ensure that 95% of all PTVs received the prescription doses.

**Table 1:** Treatment planning goals for head‐and‐neck, brain, prostate with pelvic lymph nodes, and spin SBRT cases

Head‐and‐Neck			Brain		
Organ name	Endpoint	Goal	Organ name	Endpoint	Goal
PTV_7000	V70 Gy	>95%	PTV_6000	V60 Gy	>95%
PTV_5600	V56 Gy	>95%	PTV_5940	V59.4 Gy	>95%
Brainstem	D0.03cc	<54 Gy	PTV_5100	V51 Gy	>95%
Brainstem	V30 Gy	<50%	PTV_5040	V50.4 Gy	>95%
Spinal_cord	D0.03cc	<45 Gy	Brainstem	D0.03 cc	<60 Gy
Parotid_L	Dmean	<26 Gy	OPTIC_NRV_L	D0.03 cc	<55 Gy
Parotid_R	Dmean	<26 Gy	OPTIC_NRV_R	D0.03 cc	<55 Gy
Larynx	Dmean	<35 Gy	GLOBE_L	D0.03 cc	<50 Gy
Mandible	D0.03cc	<75 or 65 Gy	GLOBE_R	D0.03 cc	<50 Gy
Trachea	Dmean	<45 Gy	LENS_L	D0.03 cc	<7 Gy
Esophagus	Dmean	<50 Gy	LENS_R	D0.03 cc	<7 Gy
Lips	Dmean	<20 Gy	CHIASM	D0.03 cc	<56 Gy
Oral cavity	Dmean	<35 Gy	COCHLEA_L	Dmean	<45 Gy
Submadibular_L	Dmean	<39 Gy	COCHLEA_R	Dmean	<45 Gy
Submadibular_R	Dmean	<39 Gy	Spinal_cord	D0.03 cc	<56 Gy

Prostate +Pelvic Lymph nodes			Spine SBRT		
Organ name	Endpoint	Goal	Organ name	Endpoint	Goal
PTV_7000	V70 Gy	>95%	Tumor_1800	V18 Gy	>90%
PTV_6200	V62 Gy	>95%	Tumor_1600	V16 Gy	>90%
PTV_4500	V45 Gy	>95%	Spinal_cord	D0.03 cc	<14 Gy
BLADDER	V63 Gy	<10%	Spinal_cord	V1000	<10%
BLADDER	V54 Gy	<20%	Sacrum/Thecal sac)	D0.03 cc	<16 Gy
RECTUM	V63 Gy	<10%	Sacrum (Thecal sac)	V1200	<10%
RECTUM	D10 cc	<63 Gy	Esophagus	D0.03 cc	<15.4 Gy
RECTUM	V45 Gy	<50%	Esophagus	V1190	<5cc
FEMORAL HEAD_L	V50 Gy	<5%	Kidney_L	V1060	<2/3
FEMORAL HEAD_R	V50 Gy	<5%	Kidney_R	V1060	<2/3
PENILE BULB	Dmean	<52.5 Gy			

**Table 2:** The same Beam configurations used for the manual plan and advanced plans

	Head Neck	Brain	Prostate	Spine
Gantry	CCW: 182‐178 CW: 178‐182	CCW: 182‐178 CW: 178‐182	Arc1: 182‐172 Arc2: 178‐182 Arc3: 200‐270 Acr4: 160‐90	Step and shoot: 280, 255, 230, 205, 181, 155, 130, 105, 80
Collimator angle	10° or 20^o^	330° or 30^o^	90^o^	0^o^
MLC leave width	0.25 cm or 0.5 cm	0.4 cm or 0.5 cm	0.4 cm or 0.5 cm	0.25 cm or 0.5 cm
Energy	6 MV	6 MV	10 MV	6 MV

### 2.2 Three advanced planning tools

The automatic planning (AP) tool developed by the Pinnacle treatment planning system (version 9.10, Philips Radiation Oncology Systems, Fitchburg, WI), is designed to mimic the manual process by automatically adjusting and adding planning objectives progressively and iteratively.^8^ The research plans generated for this study did not have additional manual optimizations after AP planning. The AP planning tool has been clinically implemented at Cleveland Clinic .

The knowledge‐based planning (KBP) is implemented in the Eclipse treatment planning system dedicated for research (version 13.6, Varian, Palo Alto, CA), which hosts anonymous patient cases and is separate from clinic patient database. KPB planning uses the dose and volume endpoints achieved from previous IMRT plans as the planning objectives for the new patient with a similar anatomic site. More specifically, using machine learning algorithms, a cancer‐specific model can be built based on the training dataset which is composed of anatomic and dosimetric information of prior patients. In the current study, all the KBP models are trained and validated with one institution's datasets, outside the 20 cases used in this study. The research plans generated from KBP were not further tuned to improve plan quality after the plans were submitted for comparison. The KBP planning tool has been clinically implemented at Duke University.

The multi‐criteria optimization (MCO) is implemented in the RaySearch planning system (version 4.9.9, Stockholm, Sweden). Rather than generating a single plan, the Raysearch planning system using MCO creates a series of Pareto optimal plans for a specific case.^14^, ^15^, ^17^ The advantage of MCO planning is that the plan trade‐offs are visible to physicians and planners and allow them to evaluate these plans and then select an optimal plan for each patient‐specific case. The research plans generated from MCO were not further optimized after the plans were submitted for comparison. The MCO planning tool has been clinically implemented at Akron General Hospital.

### 2.3 Plan evaluation

The plan quality evaluation was performed based on quantitative dosimetric parameters extracted from the plans, including percentage of dose coverage on target volumes, the maximum or mean dose of the OARs, the conformity index (CI), and the homogeneity index (HI). The CI is defined as

(1) $$CI=V_{\mathrm{Rx}}/V_{\mathrm{PTV}}$$

where V_Rx_ is the tissue volume covered by the prescription dose for the PTV (high dose PTV, if there are multiple PTVs) and V_PTV_ is the volume of the corresponding PTV. For the ideal case, CI equals to 1. The HI is defined as

(2) $$HI=D_{\max}/D_{\mathrm{Rx}}$$

where D_max_ is the maximum dose to 0.03 cc and D_Rx_ is the prescription dose for the high dose PTV (HD‐PTV). In addition, the total monitor units (MUs) per plan were also used to assess the delivery efficiency.

For each site, we plotted box plots of the PTVs and OARs for the four types of plans. In addition, we randomly selected one case from each site to compare dose distributions and dose volume histograms among the four plans case by case.

## 3 RESULTS

### 3.1 HN cases

**Figure 1:**
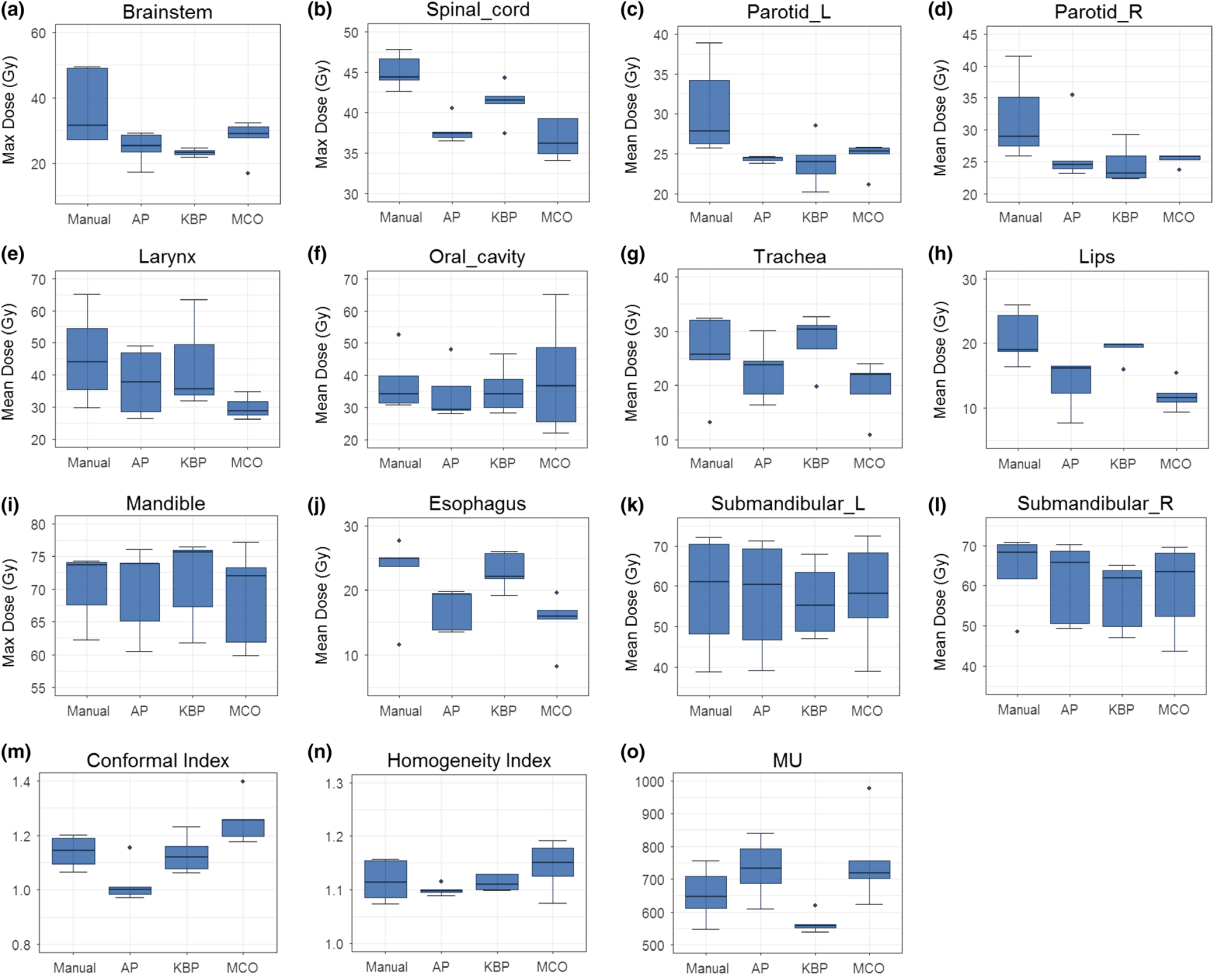
Comparison of the dosimetric performance between the three advanced plans and manual plans in head‐and‐neck cases for maximum dose for brainstem (a) and spinal cord (b), mean dose for parotid glands (c and d), larynx (e) and oral cavity (f), trachea (g), lips (h), esophagus (j), and submandibular (k and l), and max dose to mandible (i) as well as conformity index (m), homogeneity index (n), and MU (o).

Figure 1 shows box plots of defined endpoints of the five head‐and‐neck cases. All advanced plans had equal or lower maximum doses to the spinal cord and brainstem, equal or lower mean dose of both parotid glands than those of manual plans [Figs. 1(a)‐1(d)]. Although the maximum doses of the brain stem and spinal cord in the manual plans were below the tolerance dose, the additional dose reductions from advanced plans could benefit if re‐irradiation is needed. For these OARs (spinal cord, brainstem, and both parotid glands), all three advanced plans showed narrow ranges on the defined dosimetric endpoints when compared to manual plans, indicating a higher consistency in plan quality. Furthermore, except for one KBP plan, all advanced plans had lower mean dose to the larynx when compared to those from the manual plans [Fig. 1(e)]. Except for one MCO plan, the mean dose to the oral cavity from the advanced plans was also lower than those from the manual plans, albeit the reductions were small [Fig. 1(f)]. Figure 1(m) and 1(n) compared plan conformal indices and homogeneity indices among advanced plans and five manual HN plans. The plan conformity indices from AP and KBP plans were equal to or better than those of manual plans, but conformity indices from five MCO plans were slightly worse than those of the manual plans [Fig. 1(m)]. Plan homogeneity indices from AP and KBP plans were equal to or better than the manual plans but homogeneity indices from MCO plans were worse than those of manual plans, indicating trade‐offs among the OAR sparing, plan conformity, and homogeneity [Fig. 1(n)]. The MUs from AP and MCO plans were slightly higher than those of manual plans and KBP plans had the lowest MUs when compared to the manual, AP, and MCO plans [Fig. 1(o)].

**Figure 2:**
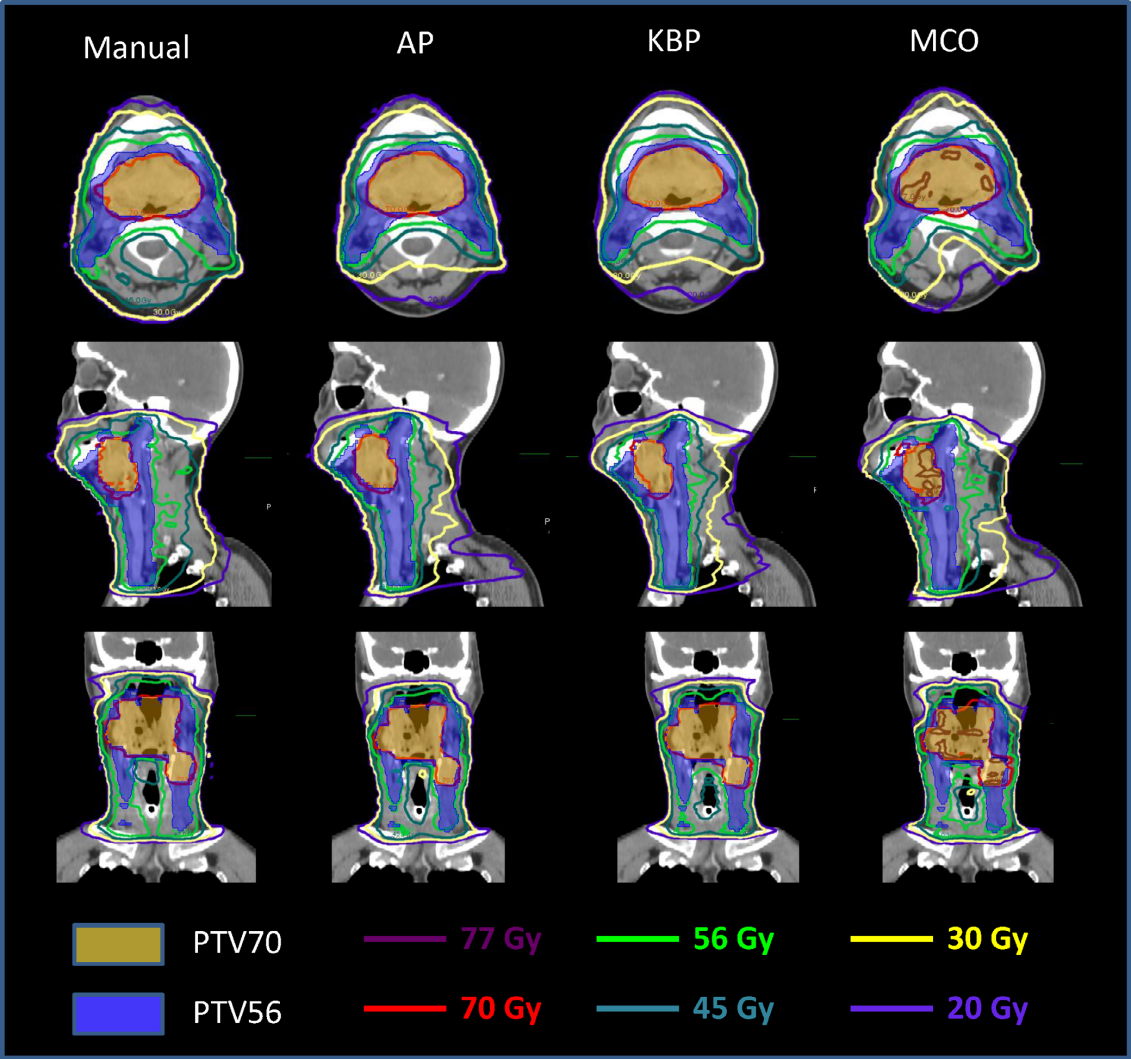
Representative isodose distributions from Manual, automatic planning (AP), knowledge‐based planning (KBP), and multi‐criteria optimization (MCO) plans for a head‐and‐neck patient.

**Figure 3:**
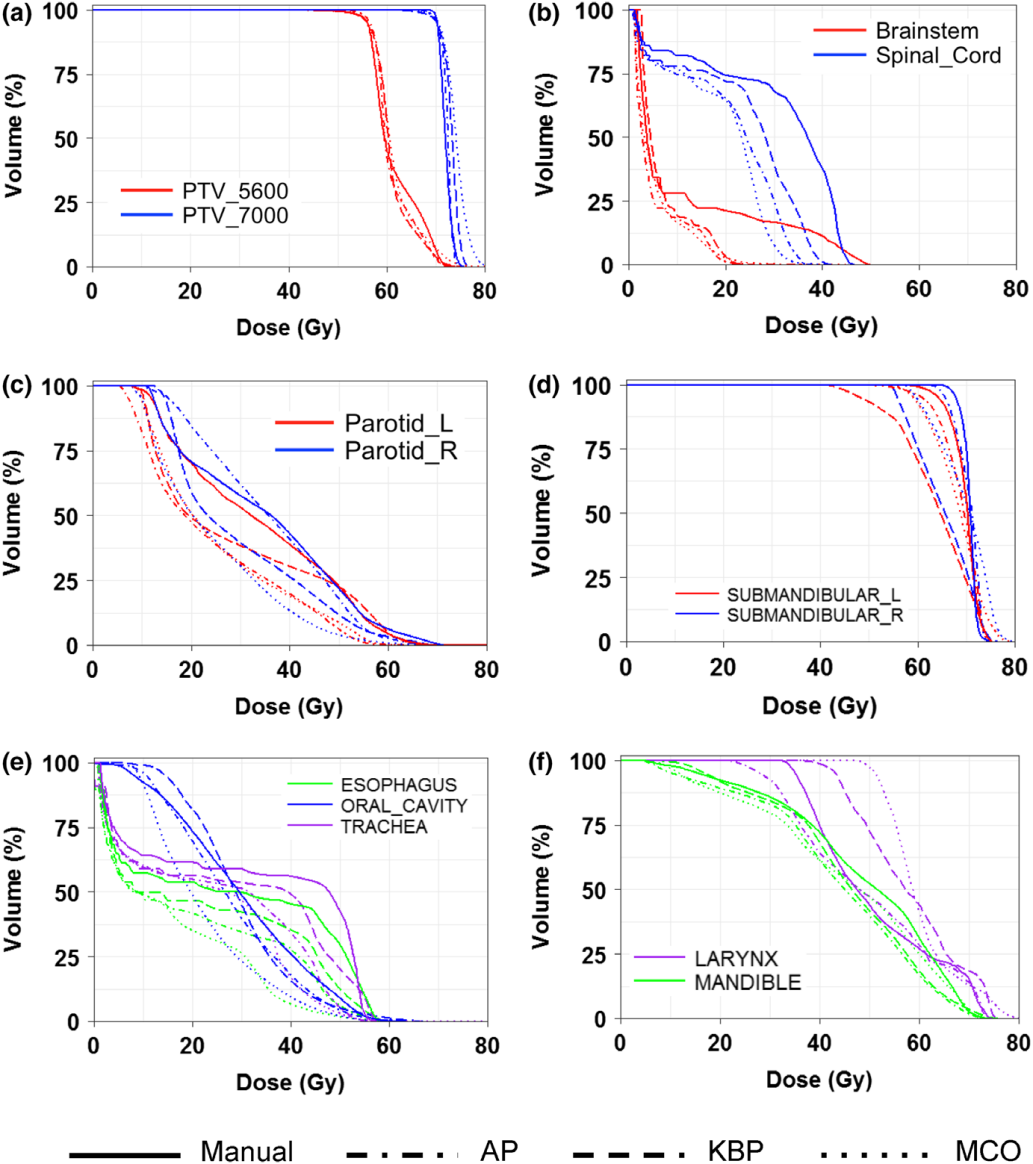
Representative DVHs of a head‐and‐neck patient for all four planning methods for tumor targets (a) and organs‐at‐risk (OARs), such as brainstem and spinal cord (b), parotid glands (c), submandibular (d), esophagus, oral cavity, trachea (e), and larynx, mandible (f).

Figure 2 shows representative isodose distributions for a randomly selected HN case. Compared to the manual plan, the advanced plans showed more conformal dose distributions, especially in the middle to low dose ranges (for instance, the 45 and 30 Gy isodose lines in Fig. 2). In addition, noticeable hotspots were observed in the HD‐PTV with the MCO plan. Fig. 3 shows the DVHs for all four methods for the same HN case as in Fig. 2. The AP and the manual plans achieved similar PTV homogeneity, while KBP and MCO plans were less homogenous with long tails noted in the HD‐PTV DVHs. In addition to equal or better dose sparing to the spinal cord, brain stem, and parotid glands [Figs. 3(b) and 3(c)], advanced plans achieved better sparing for both left and right submandibular, esophagus, and trachea when compared to the manual plan [Figs. 3(d) and 3(e)]. The maximum dose to the mandible was similar from all plans [Fig. 3(f)]. However, for the larynx, KBP and MCO resulted in worse DVHs compared to AP and manual plans, indicating the trade‐off between larynx sparing and other OARs' sparing for KBP and MCO plans for this typical case [Fig. 3(f)].

### 3.2 Brain cases

**Figure 4:**
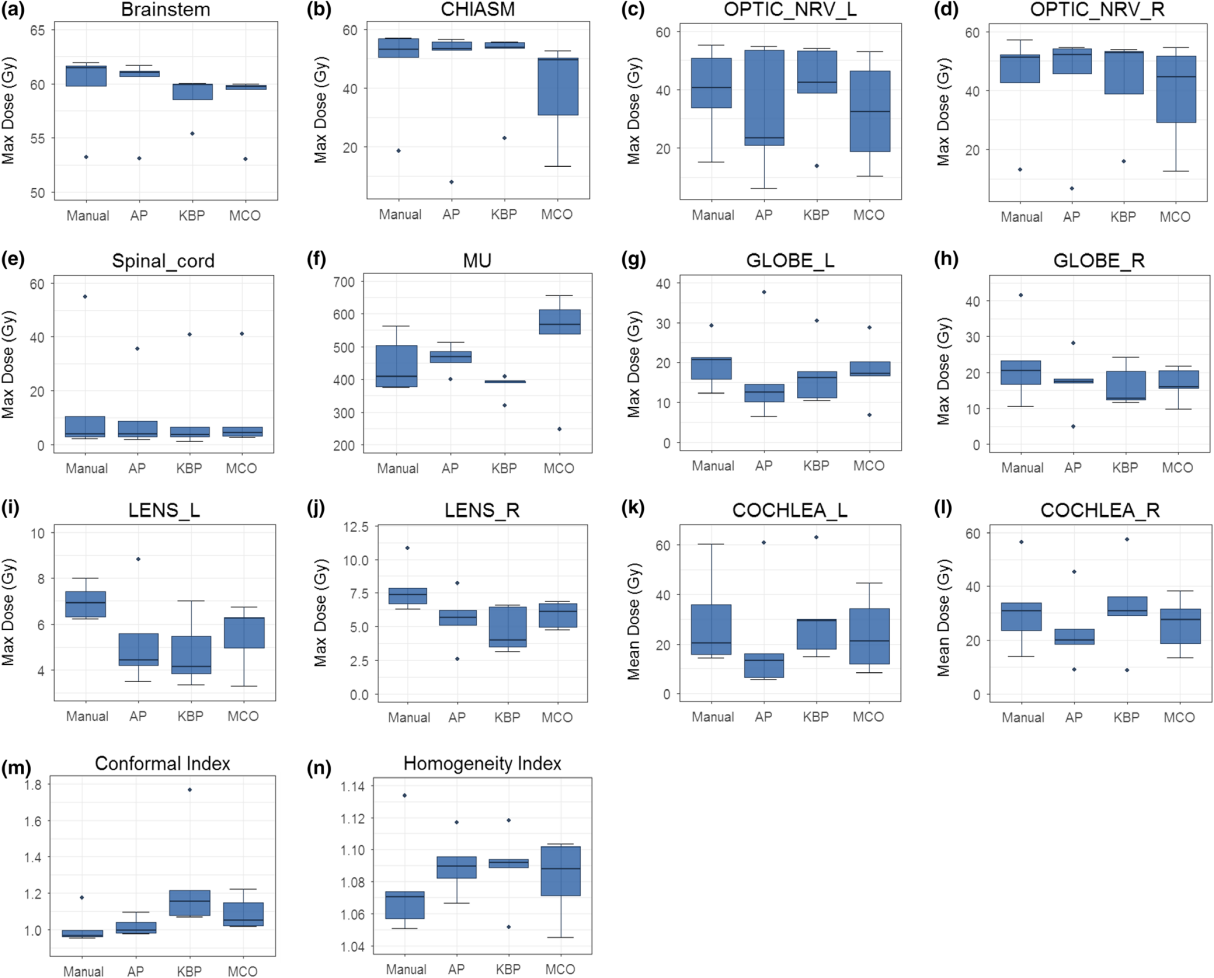
Comparison of the dosimetric performance between the three advanced plans and manual plans in brain cases for maximum dose to brainstem (a), chiasm (b), optical nerves (c and d), spinal cord (e), globes (g and h), and lens (i and j), mean dose to cochlea (k and l), as well as conformity index (m), homogeneity index (n), and MU (f).

Figure 4 shows box plots of defined endpoints of the five cases in this treatment site. For these cases, the maximum doses to the brainstem and chiasm are limiting doses. The maximum dose to the brain stem from the AP plans are comparable to those from the manual plans, exceeding or approaching the dose limit of 60 Gy while the KBP and MCO plans meet or lie below the dose limit [Fig. 4(a)]. The maximum doses to the chiasm are comparable among manual, AP, and KBP plans while the maximum doses to the chiasm from MCO plans are substantially lower than those from the manual plans [Fig. 4(b)]. The results of the maximum doses to optic nerves are mixed among four types of plans [Figs. 4(c) and 4(d)]. In general, the advanced plans had equal or lower maximum dose to the optic nerves than those of the manual plans. For the spinal cord, all three advanced plans achieved lower maximum dose with narrow ranges, indicating a higher consistency in plan quality [Fig. 4(e)]. For other normal structures including eyes, lens, and cochlea, the three advanced plans had equal or lower mean doses than those of manual plans [Figs. 4(g)–4(l)]. Plan conformal indices and homogeneity indices are important for these cases. Advanced plans are less conformal and less homogenous than the manual plans, indicating a trade‐off among the OAR sparing, plan conformity, and homogeneity [Figs. 4(m)–4(n)]. The MUs from KBP plans were the lowest and the MCO plans had the highest MUs in these plans [Fig. 4(f)].

**Figure 5:**
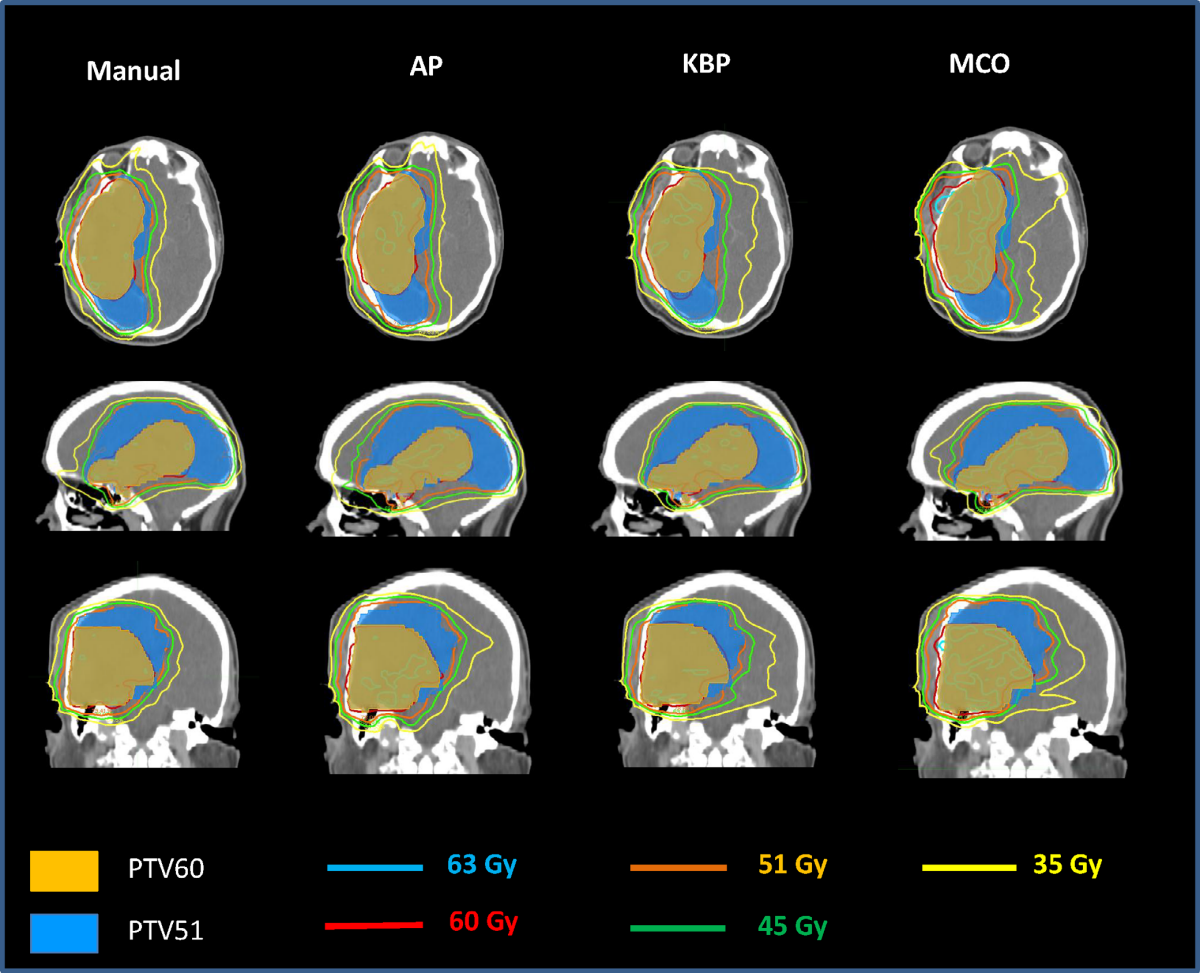
Representative isodose distributions from Manual, automatic planning (AP), knowledge‐based planning (KBP), and multi‐criteria optimization (MCO) plans for a brain patient.

**Figure 6:**
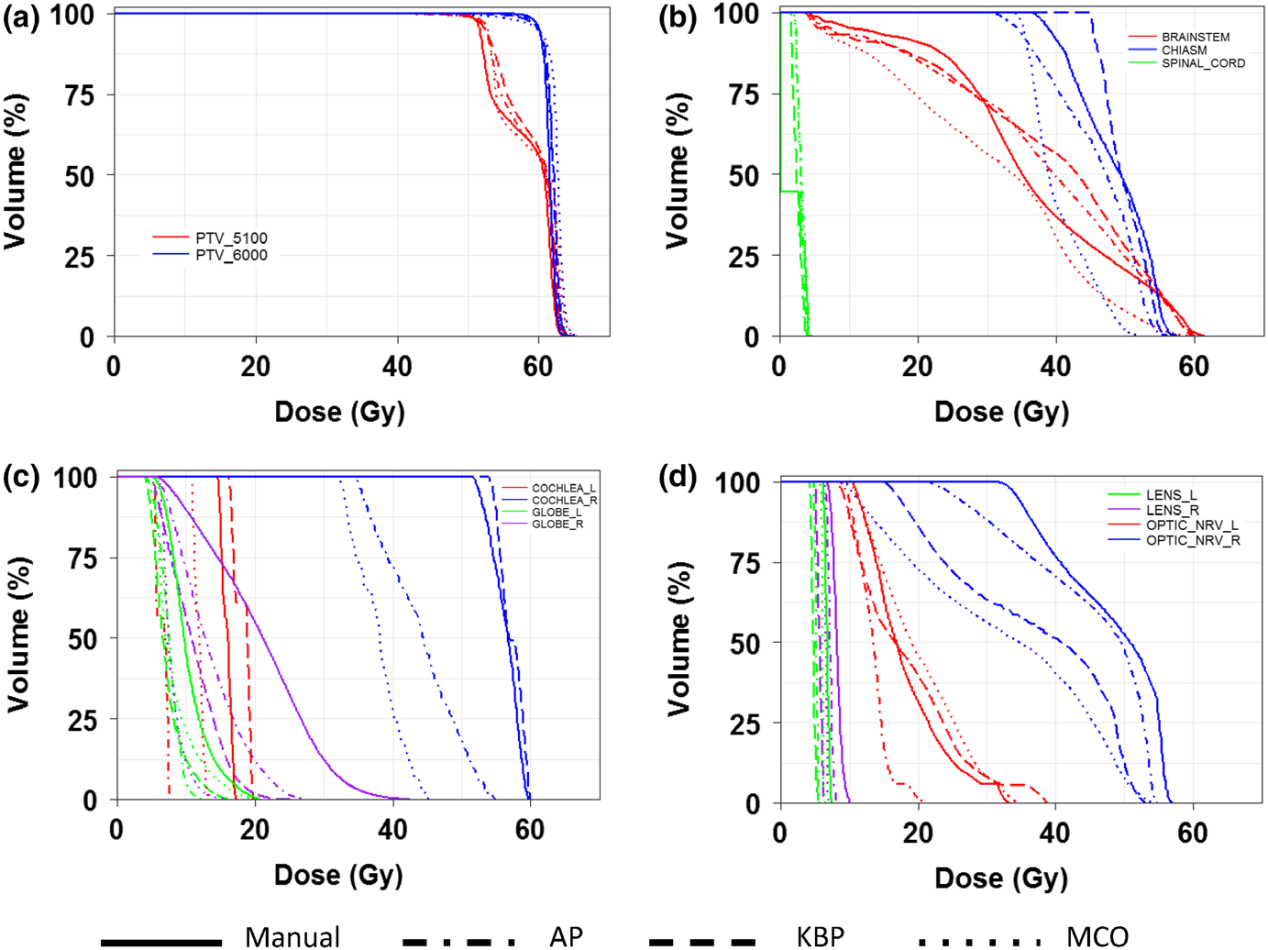
Representative DVHs of a brain patient for all four planning methods for tumor targets (a) and organs‐at‐risk (OARs), such as brainstem, chiasm, spinal cord (b), cochlea, globes (c), lens, and optical nerves (d).

Figure 5 shows representative isodose distributions for a randomly selected brain case. It showed that AP plan was slightly hotter than the manual plan with better sparing to the right eye. However, the 35 Gy isodose lines from the KBP and MCO plans were extended further to the contralateral brain. Figure 6 shows the DVHs for the same brain case as in Fig. 5. In this specific case, except for further decreasing doses to the eyes and lens using advanced plans, the quality of manual plan and advanced plans were comparable, which was reasonably balanced among plan conformity, homogeneity, and spring critical structures (i.e., brain stem and chiasm).

### 3.3 Prostate and pelvic lymph nodes cases

**Figure 7:**
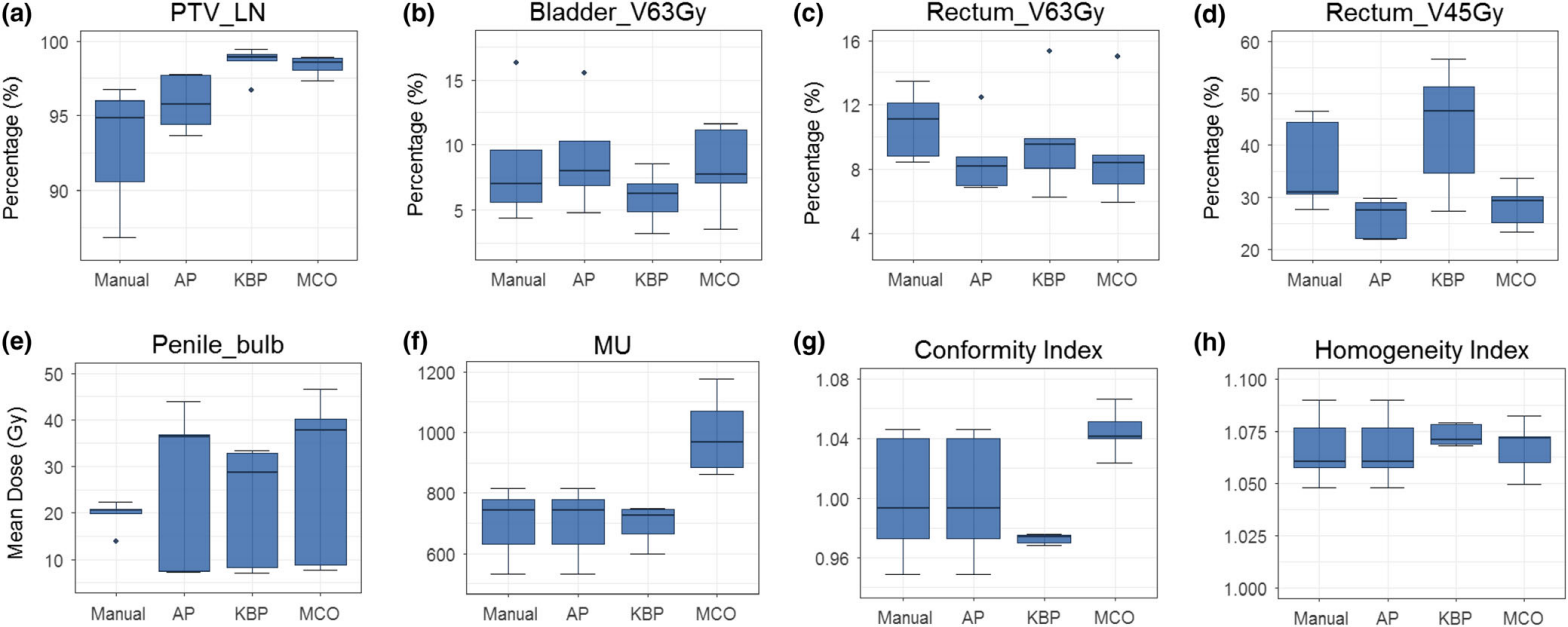
Comparison of the dosimetric performance between the three advanced plans and manual plans in prostate cases for percentage of volume for lymph node target (a), bladder V63Gy (b), rectum V63Gy (c), rectum V45Gy (d), mean dose to penile bulb (e), as well as conformity index (g), homogeneity index (h), and MU (f).

Figure 7 shows box plots of four planning methods based on defined dosimetric endpoints of the five cases in the prostate treatment site. These patients were treated with 70 Gy in 25 fractions to the prostate while concurrently treating 60 or 56 Gy to the seminal vesicle, and 50.4 or 45 Gy to the pelvic lymph nodes. The dose coverage to the prostate met the goal for all plans; the dose coverage to the seminal vesicle and pelvic lymph nodes for advanced plans met the goal while the manual plans were 1–2% below the goal for several cases [Fig. 7(a)], and this relaxed dose coverage allowed manual plan to achieve better mean dose to the penile bulb than the advanced plans [Fig. 7(e)]. For the rectum, advanced plans achieved decreased V63 Gy when compared to those of manual plans [Fig. 7(c)]. However, V45 Gy of the rectum in KBP plans was higher than those in the manual plans, which is likely due to the fact that this dosimetric endpoint was not included in the KBP model [Fig. 7(d)]. The conformity and homogeneity indices in advanced plans were comparable to those in manual plans with slightly worse conformity indices in MCO plans [Figs. 7(g) and 7(h)]. The total MUs were higher in MCO plans, indicating higher modulations in these plans [Fig. 7(f)].

**Figure 8:**
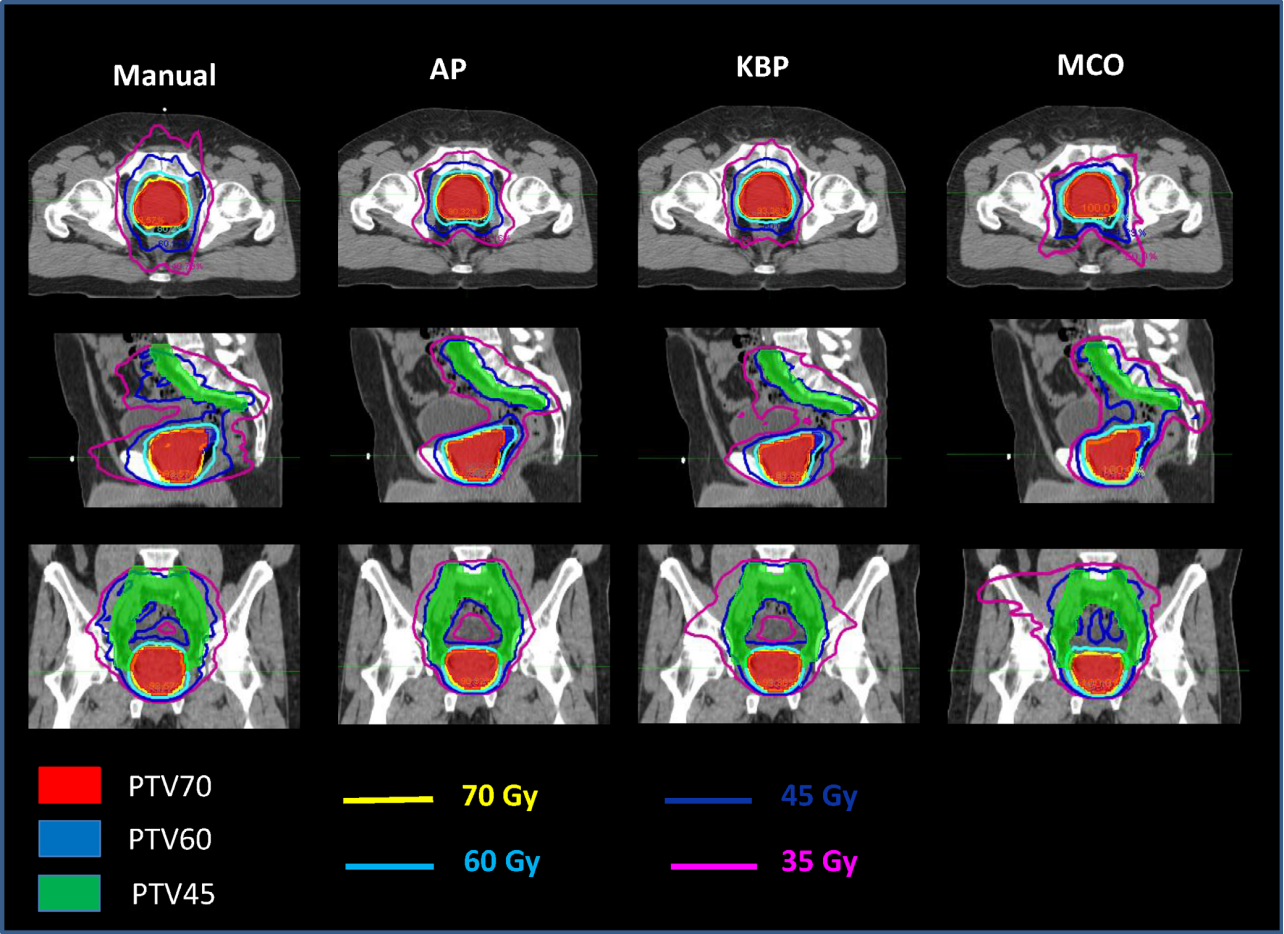
Representative isodose distributions from Manual, automatic planning (AP), knowledge‐based planning (KBP), and multi‐criteria optimization (MCO) plans for a prostate patient.

**Figure 9:**
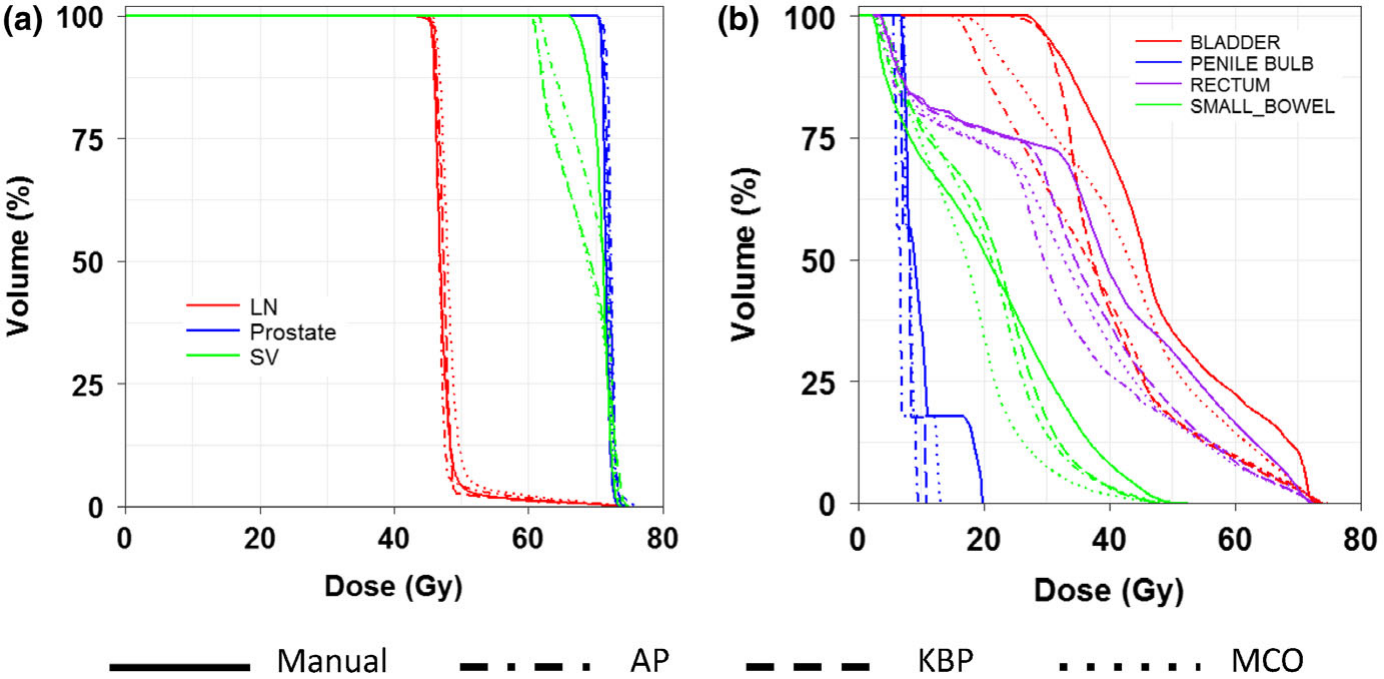
Representative DVHs of a prostate patient for all four planning methods for PTV targets (a) and organs‐at‐risk (OARs), such as bladder, rectum, small bowel, and penile bulb (b).

Figure 8 shows the representative isodose distributions for a randomly selected prostate case. For this particular case, AP and KBP were more conformal to the pelvic lymph nodal volume than the manual and MCO plans. At the low dose level of 35 Gy, AP plan showed the best control on low dose spillage. As see in Fig. 9, the DVHs of the rectum and bladder were improved in three advanced plans when compared to the manual plan.

### 3.4 Spine SBRT cases

**Figure 10:**
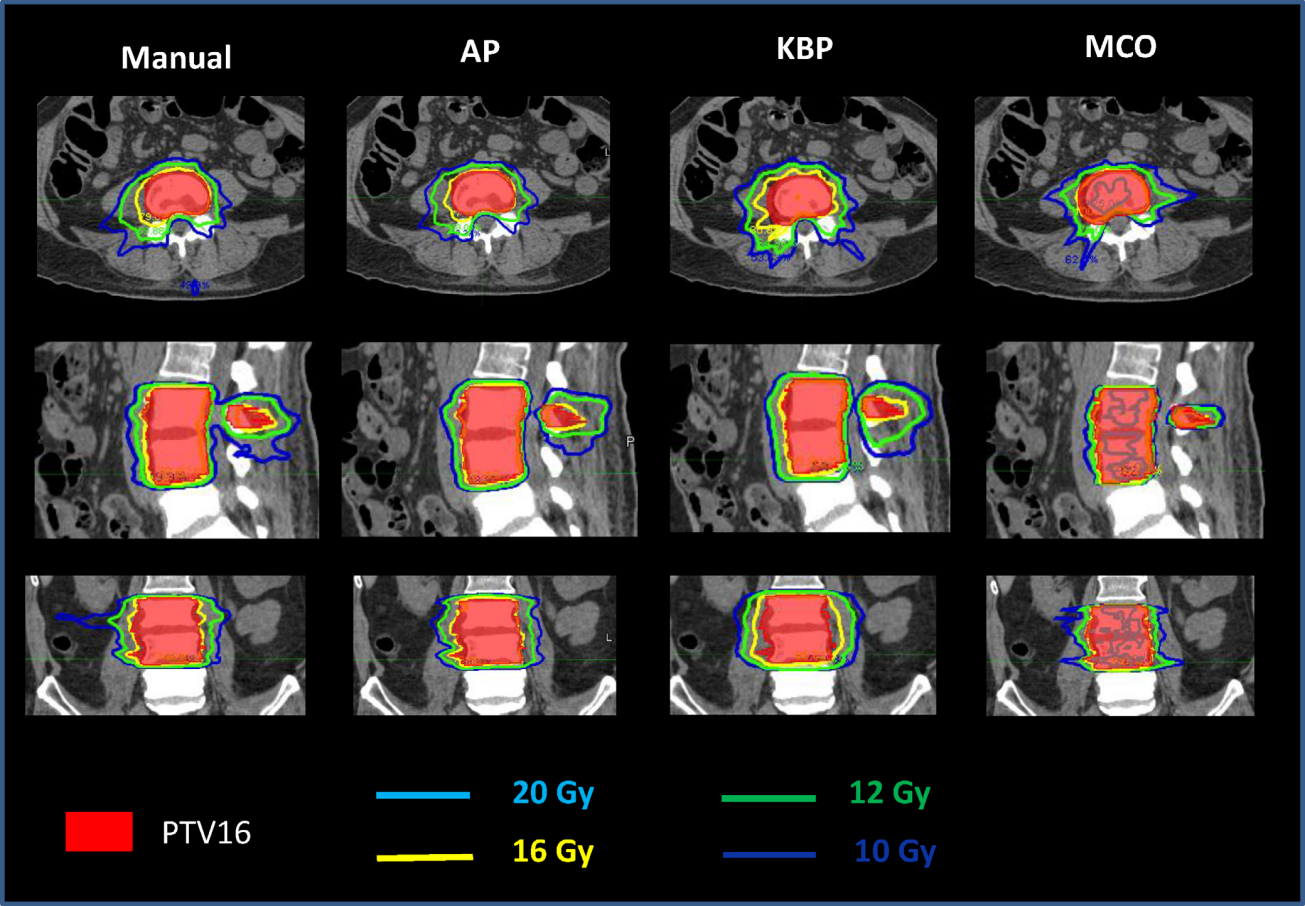
Comparison of the dosimetric performance between the three advanced plans and manual plans in spine cases for maximum dose to the spinal cord (a) and spinal cord V10Gy (b) as well as conformity index (c), homogeneity index (d) and MU (e).

**Figure 11:**
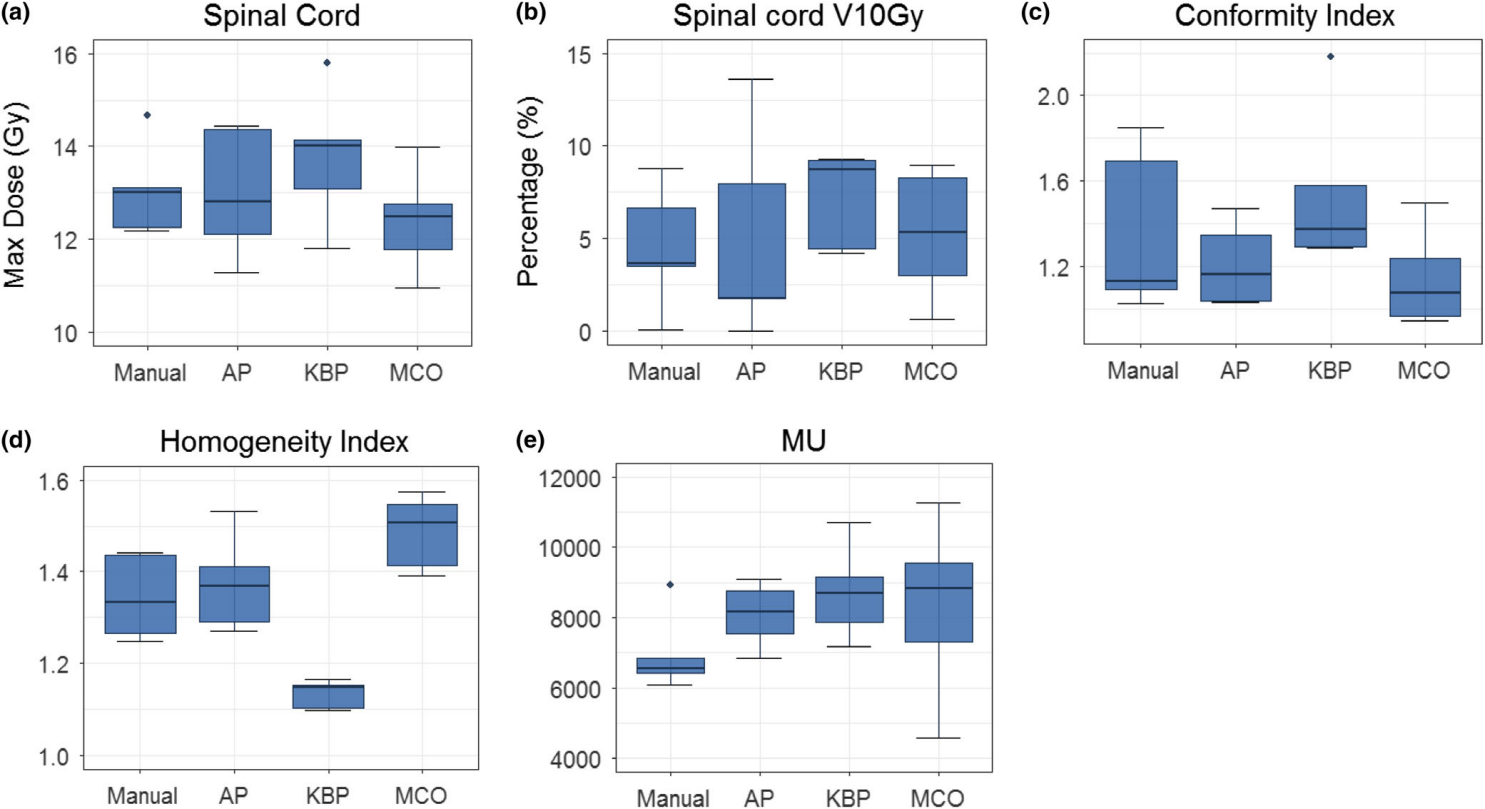
Representative DVHs of a spine patient for all four planning methods for PTV and spinal cord.

**Figure 12:**
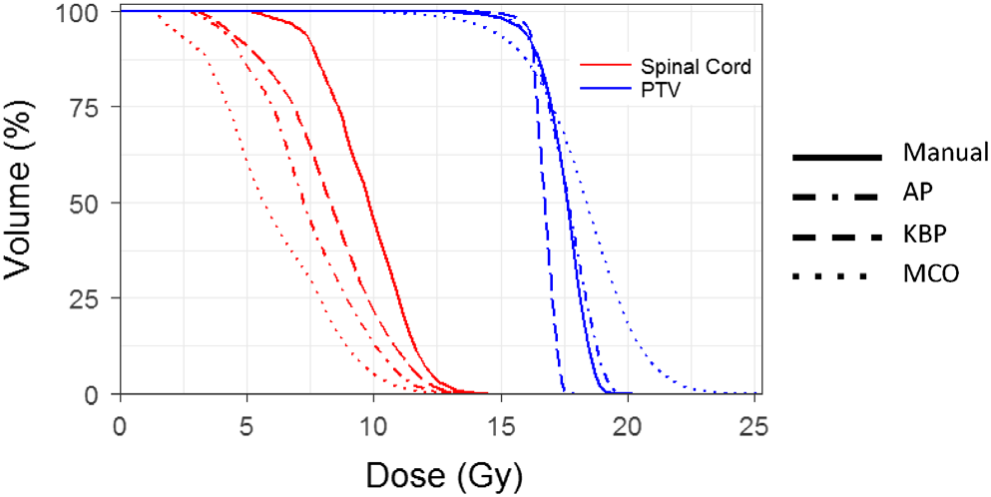
Representative isodose distributions from Manual, automatic planning (AP), knowledge‐based planning (KBP), and multi‐criteria optimization (MCO) plans for a spine patient.

The spine cases were treated with a prescription dose of 18 or 16 Gy, located at T11, L1, L3‐4, C4, and T3–T4. Figure 10 shows the box plots of four planning methods based on defined endpoints of the five spine cases. The MCO plans achieved the lowest maximum doses to the spinal cord and the KBP plans had the highest maximum dose to the spinal cord [Fig. 10(a)]. The advanced plans had worse V10 Gy to the spinal cord than the manual plans [Fig. 10(b)]. All KPB plans were most homogeneous with trade‐off of the least conformity, while all MCO were most conformal with trade‐off of least homogeneity [Figs. 10(c) and 10(d)]. MCO plans again, have the highest MUs among all plans [Fig. 10(e)]. Figure 11 and 12 show representative isodose distributions and DHVs for a randomly selected spine SBRT case. For this particular case, the MCO plan achieved the lowest maximum dose to the spinal cord and most conformity but with trade‐off of the least plan homogeneity**.**

## 4 DISCUSSION

To our knowledge, this is the first side‐by‐side comparison of three advanced treatment planning tools with the manual plans. Three institutions participated the study, and each has implemented one of the advanced planning tools clinically. In the current study, the advanced plans from these institutions, however, did not undergo multiple iterative processes as the clinical plans often did. For the research purpose, these advanced plans were created by simply applying the advanced tools with one or two attempts. Therefore, the quality of these research plans did not undergo the clinical evaluation process, specifically physicians' evaluation as the manual plans.

Our results indicated that advanced planning methods are beneficial especially for the complex plans such as head‐and‐neck cancer. Due to the complexity of the tumor shape and location and more involved OARs, it is difficult to take all the planning objectives into account when manually optimizing a plan. Considering substantial variation between cases, the small sample size of five cases may not have enough power to evaluate the statistical difference between the advanced plans and manual plans, however, the trend of improvement for normal tissue sparing is clearly notable for each HN plan. For other disease sites, such as prostate and spine that contain fewer OAR, the advanced plans exhibit similar dose distribution and normal tissue sparing as compared to the manual plans. One possible reason is that both spine and prostate cases contain fewer OAR and tumor targets, and hence it is easier for human planners to achieve a clinically optimized plan with inverse planning tasks. Another possible reason is that there is no further optimization after one round of automatic optimization for the advanced planning methods.

Many previous studies have demonstrated promising results of improvement for OAR sparing using AP. For example, using ten challenging HN cases, Gintz et al. demonstrated that AP can provide lower OAR doses compared to human‐driven VMAT plans despite less homogeneous dose distributions in AP plans.^18^ Hansen et al. performed a prospective study, in which three senior oncologists blindly compared AP and manual plans and picked 29 out of 30 plans because of better homogenous dose distributions and significantly lower OAR doses without compromising the target coverage.^7^ In the current study, we did not further optimize the plan after initial automatic process. In contrast, all the previous work performed postoptimization after the automatic step, which included adding more objectives or adjusting weights of the objectives if violations of initial constraints for target or critical organs were found. Without postoptimization our AP plans may not reach the best dose sparing for OARs. As reported by Hazell et al., better OAR sparing was obtained with slightly compromising target coverage relative to the manual plans due to lack of the postoptimization.^8^

The concept of KBP can be broad, including early attempts of using class solutions,^19^, ^20^, ^21^ applying template‐based planning objectives,^22^, ^23^ and model‐based predictive planning objectives.^10^, ^12^, ^13^ The model‐based KBP builds a model that is trained by prior clinically optimal plans from a specific treatment site to predict patient‐specific achievable planning objectives. With the benefit of having patient‐specific achievable planning objectives, the dependence on planner to estimate the optimal planning objectives is largely reduced or eliminated, thus interplanner and interinstitution plan quality variations can be reduced.^10^ It is worthwhile noting that several aspects can affect the accuracy of the model and consequently the precision of the predicted objectives. For instance, the number of cases available for the model training, the variation range of the anatomy, and the quality of the plans, can all affect the model prediction accuracy.^24^, ^25^

MCO provides planners insight into the lowest achievable OAR dose, and navigated trade‐offs to allow physicians to be more involved in the treatment plan decision, which make interaction among treatment planning systems, planners, and physicians more efficient.^16^, ^26^, ^27^ Since the MCO created plans which are all located on the Pareto surface, obviously unacceptable plans were excluded from this dataset, and transiting from one plan to another relies on the priority of OAR sparing based on the patient‐specific situation. Hong et al. reported that MCO is suitable for patient with pancreatic cancer and helps physician select a favorite plan quickly.^26^ Craft et al. showed that MCO planning time was dramatically shorter than the clinical standard planning time.^27^

One advantage of these advanced plans is that the plan quality variation is much less than manually optimized plans and interplanner variation is reduced.^8^ For the manual plan, it is difficult to predict to what extent an OAR can be spared before executing the optimization, and the population‐based guideline was generally set either loose or tight initially. The amount that can be modified in the subsequent planning process depends on the experience of the planner or the clinical turnaround time available. Those advanced planning methods showed potential of improving the plan quality with better dosimetirc performance than manual methods with less variability, which can help transfer planning expertise to the clinical practice where large plan variation exists.^28^ In addition, the advanced planning methods are not limited to one modality of treatment and can be generalized to other delivery techniques such as TomoTherapy® (Accuray, Inc., Sunnyvale, CA) and Gamma Knife (Elekta, Stockholm, Sweden).^29^, ^30^

One limitation of the current study is the small sample size. Considering the complexity of this collaborative work involving three institutions with expertise in different advanced treatment planning systems, only 20 cases were included in this study. While the sample size is small, this multi‐institutional study provides valuable assessment of clinical usage for three important advanced treatment planning tools across four different disease sites. Another limitation of the study is that the manual plans were generated by experienced dosimetrists and, therefore, no substantial reduction of variation in the plans created by the advanced planning methods was observed compared to typical manual plans, which imply that the advanced plans are less dependent on the experience and skills of planners, and able to provide more consistent plans.

## 5 CONLUSION

Advanced treatment planning tools, including auto‐planning, knowledge based planning, and multi‐criteria optimization, can assist planners to create a better or equivalent plan quality compared to manual plans. These tools are more beneficial for complex cases such as HN patients with multiple PTVs, or cases where critical structures are spared. For less complex cases, the advanced plans showed comparable dosimetric endpoints with the manual plans.

## CONFLICT OF INTERESTS

The authors received research support from Philip Medical Systems and Varian Master Research Agreement.
